# Benign acute children myositis: 5 years experience in a tertiary care pediatric hospital

**DOI:** 10.1007/s00431-023-05115-9

**Published:** 2023-07-18

**Authors:** Federica Attaianese, Andrea Costantino, Cristiana Benucci, Donatella Lasagni, Sandra Trapani

**Affiliations:** 1 Department of Health Sciences, University of Florence, Florence, Italy; 2 Pediatric Unit, Meyer Children’s Hospital IRCCS, Viale Pieraccini 24, Florence, 50139 Italy

**Keywords:** Benign myositis, Children, Pediatrics, Myalgia, Gait complaint, Influenza

## Abstract

Benign acute childhood myositis (BACM) is a self-limited childhood illness, and viral infections mainly cause it. Clinical and laboratory alterations usually normalize rapidly; generally, the only medical intervention required is supportive (hydration and analgesic medication). The low awareness about BACM often led to delayed diagnosis and unneeded ancillary investigations. This study aims to better characterize the clinical and laboratory features of BACM to improve the diagnostic process and inpatient and outpatient management. We conducted a retrospective study selecting all children admitted to Meyer’s Children’s Hospital-IRCCS (Florence, Italy) with a diagnosis of BACM over the last 5 years, both those visited at Emergency Department (ED) and those admitted to the Pediatric Unit. Clinical, laboratory, and instrumental data were collected from electronic clinical records and analyzed. Overall, sixty-five patients were enrolled; 49 children were visited and discharged directly from ED, whereas 16 were admitted in the Pediatric or Neurologic Wards. The median age was 6.56 years (IQR 4.9–9.1). Male gender (66.1%) and Caucasian ethnicity (70%) were prevalent. Most patients were admitted during winter, and a second peak was found in autumn. All patients had bilateral calf pain, most of them (87.7%) associated with asthenia and refuse to walk (93.8%). Prodromal symptoms were fever (75.3%), cough (32.3%), coryza (26.1%), sore throat (26.1%), and vomiting (15.3%). The median value of CPK was 1827 U/L (IQR 915.5–2462) at peak. CPK median time to normalization was 7 days (IQR 7–8.5) from the nadir. Influenza B was the virus most frequently BACM associated, followed by Influenza A; a novel association with Sars-CoV-2 has been detected. Two patients had pathogenic variants at the Next Generation Sequencing myopathies panel.

*Conclusion*: School-aged children admitted to the hospital with walking difficulty and myalgia, generally after an upper respiratory tract infection with a moderate CPK elevation, should remind at first of BACM. Rapid complaint resolution and biochemical markers normalization will prevent unnecessary tests and inappropriate therapies.

**Table:** 

**What is Known:** *• BACM is a self-limited syndrome associated with acute infections. Influenza A and B viruses are the main etiological agents, but BACM may be related to many other microorganisms like Parainfluenza virus, Epstein-Barr virus, Cytomegalovirus, Human herpesvirus 6, Respiratory syncytial virus, Coxsackieviruses, Mycoplasma pneumoniae, Streptococcus pyogenes, Legionella, and Salmonella spp.* • *Clinical and laboratory alterations usually normalize rapidly; generally, the only medical intervention required is supportive (hydration, analgesic medication). Evolution in rhabdomyolysis and kidney damage is possible but rarely reported.*	
**What is New:** • *Sars-CoV-2 could be an emerging possible cause of BACM. During and after the Sars-CoV-2 outbreak, virus infection seasonality has changed, and so has BACM seasonality.* • *Screening tests for muscular and metabolic disorders are recommended in recurrent myositis and/or cases with marked CPK elevation (≥ 5000 U/L).*

**What is Known:**

*• BACM is a self-limited syndrome associated with acute infections. Influenza A and B viruses are the main etiological agents, but BACM may be related to many other microorganisms like Parainfluenza virus, Epstein-Barr virus, Cytomegalovirus, Human herpesvirus 6, Respiratory syncytial virus, Coxsackieviruses, Mycoplasma pneumoniae, Streptococcus pyogenes, Legionella, and Salmonella spp.*

• *Clinical and laboratory alterations usually normalize rapidly; generally, the only medical intervention required is supportive (hydration, analgesic medication). Evolution in rhabdomyolysis and kidney damage is possible but rarely reported.*

**What is New:**

• *Sars-CoV-2 could be an emerging possible cause of BACM. During and after the Sars-CoV-2 outbreak, virus infection seasonality has changed, and so has BACM seasonality.*

• *Screening tests for muscular and metabolic disorders are recommended in recurrent myositis and/or cases with marked CPK elevation (≥ 5000 U/L).*

## Introduction

Benign acute childhood myositis (BACM) is a muscle disorder that may accompany children’s acute infections. It was first described 60 years ago by the Swedish pediatrician Lundberg as myalgia cruris epidemica [1] and may occur as an epidemic or sporadic disease. Influenza viruses have been most frequently associated with epidemic forms; among them, Influenza B appears responsible for more cases than Influenza A [2–4]. Sporadic forms are related to many other microorganisms, including Parainfluenza virus, Sars-CoV-2, Epstein-Barr virus (EBV), Cytomegalovirus, Human herpesvirus 6, Respiratory syncytial virus, Coxsackieviruses, *Mycoplasma pneumoniae*, *Streptococcus pyogenes*, *Legionella*, and *Salmonella* spp. [5, 6]. Supporting the evidence that viral illness is the most common cause, BACM tends to be prevalent in the late winter and early spring [2]. School-aged boys are more commonly affected [2, 3, 5]. Clinical manifestations may vary from mild myalgia to rhabdomyolysis. The acute onset of bilateral calves’ pain following an acute flu-like illness is the most frequent presentation. Children affected typically present a wide-based, stiff-legged gait, toe-walking, or weight-bearing refusal [3, 7–9]. The clinical examination may reveal calves’ tenderness with normal neurological findings including preserved ankle and knee reflexes. Muscle pain resolution usually occurred in less than 10 days, without residual sequelae [2, 5]. Typical laboratory alterations related to BACM are the increase of serum creatine phosphokinase (CPK) levels, aspartate aminotransferase (AST), and potassium. Other laboratory findings, like leucocytosis and C-reactive protein (CRP) increase, may be related to the acute infection. Both immune-mediated processes and direct pathogen muscle invasion are supposed to be the underlying mechanisms of benign myositis [4]. The differential diagnosis includes rheumatological disorders (juvenile rheumatoid arthritis, dermatomyositis, vasculitis), neurological diseases (Guillain-Barré syndrome, cerebellar ataxia, transverse myelitis), infectious diseases (arthritis, osteomyelitis), and others (deep vein thrombosis, fractures, malignancies). Furthermore, inherited neuromuscular diseases impairing muscular energy production should be considered following two or more attacks [2, 4, 5, 10]. BACM management consists of symptomatic and supportive treatments including intravenous hydration, to promote muscle enzyme clearance and prevent complications. Among them, rhabdomyolysis, which may result in kidney damage secondary to myoglobinuria, should be considered [4]. Concerning the sporadic forms, the low awareness about BACM often led to delayed diagnosis and unneeded ancillary investigations [5]. In this study, we aimed to better characterize the clinical and laboratory features of BACM to improve the diagnostic process and inpatient and outpatient management.

## Materials and methods

**Fig. 1:**
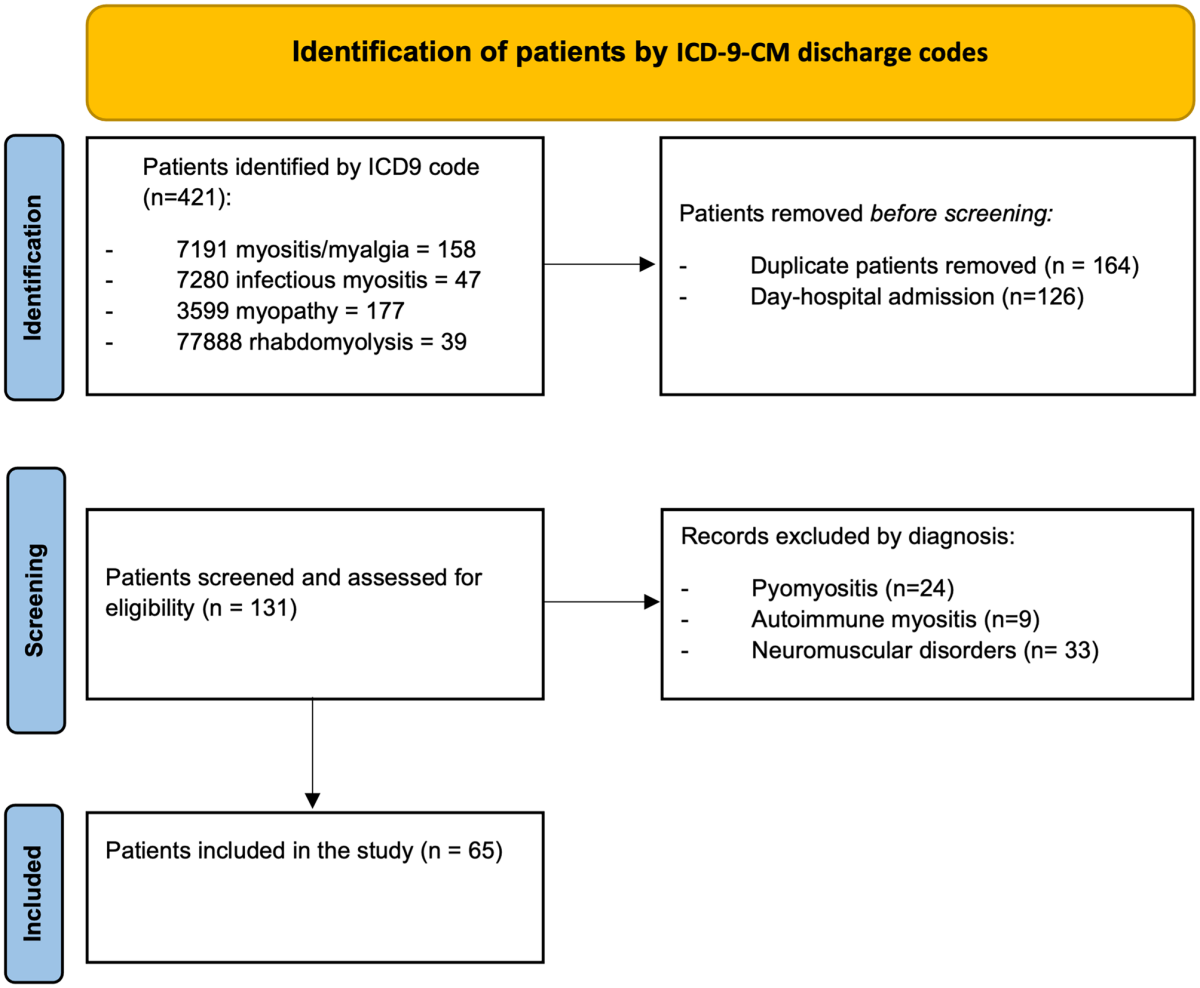
Patients’ selection flow diagram

We conducted a retrospective study on all the children admitted at the Emergency Department (ED) of Meyer’s Children’s Hospital-IRCCS (Florence, Italy) and discharged with the diagnosis of BACM, in the last 5 years (from January 2018 to March 2023), including patients discharged after the ED visit and those admitted at the Pediatric and Neurologic Wards. Patients were identified using the institutional discharge database by the following International Classification of Diseases, 9^th^ Revision, Clinical Modification (ICD-9-CM) discharge codes: 7291 myositis/myalgia, 7280 infectious myositis, 3599 myopathies, and 77,888 rhabdomyolyses. Among them, the eligible cases were screened after the exclusion of day-hospital admissions and duplicates. The medical records of the screened patients have been reviewed: cases with bacterial infectious myopathies, like pyomyositis, muscular autoimmune disorders like dermatomyositis, and chronic neuromuscular diseases were excluded. Finally, the remaining patients with clinical and/or laboratory evidence of acute infection and acute findings of serum CPK increase were included (Fig. 1). For each patient, all medical information was collected, including age, gender, the month of admission, previous intensive exercise or trauma, familial and past histories (with attention to the prodromal symptoms in the weeks before onset), clinical manifestations, management (type and duration), length of stay (LOS), time to clinical resolution, and outcome.

The laboratory findings, including inflammatory markers, complete blood cells count, CPK, lactate dehydrogenase (LDH), AST, alanine aminotransferase (ALT), urea, serum electrolytes, creatinine, and urine routine, both at admission and peak, were registered. Reference values were considered as follows: CPK 30–150 U/L, AST 5–40 U/L, ALT 5–41 U/L, LDH 10–250 U/L. Leucocytosis was defined by leucocyte count > 10.000/mm^3^, and leucopenia by the value of 4.500/mm^3^.

For each parameter, time to peak was also recorded. Etiological research, including infectious agents (real-time Polymerase-chain-reaction on blood or nasal swab) and muscular and metabolic tests, was collected when available.

Numbers and percentages were used for categorical variables, and median and interquartile ranges (IQR) were presented for non-normal distributed continuous variables.

## Results

### Epidemiology

We first screened 131 patients and then selected 65 of them (Fig. 1). All patients were admitted to the ED, 16 were hospitalized at the Pediatric or Pediatric Neurology ward, whereas the other 49 were discharged from ED within 24 h.

**Fig. 2:**
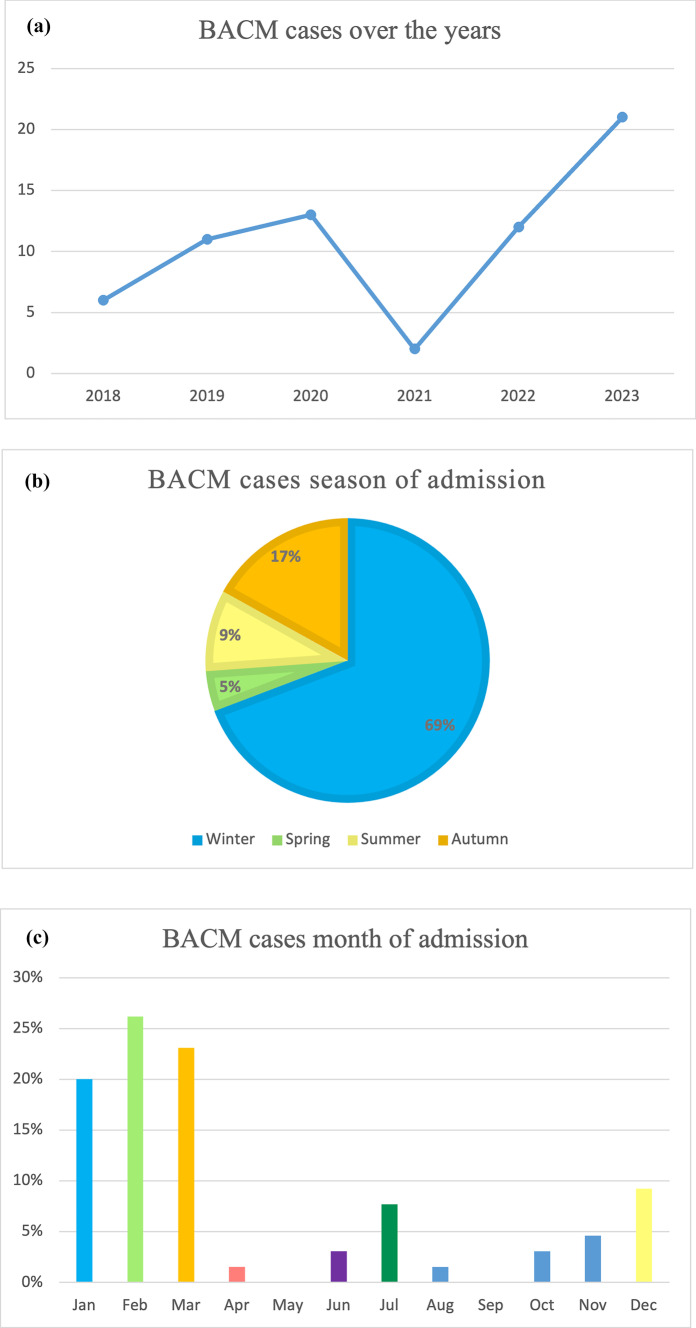
BACM cases per **a** year, **b** season, and **c** month of admission

The median age was 6.56 years (IQR 4.9–9.1). Male gender (*n* = 43, 66.15%) and Caucasian ethnicity (*n* = 45, 70%) were prevalent. Four patients had a history of previous disease, respectively diabetes type, trisomy 21, congenital hypothyroidism, and post-hemorrhagic hydrocephalus. Three patients had a history of previous myositis/hyperCKemia. Regarding annual incidence, 2023 was the year with the highest caseload. During the Italian state of emergency due to COVID-19 (21 February 2020–31 March 2022), only two cases of BACM were admitted to our hospital. Most patients were admitted during winter, with a second peak in autumn (Fig. 2 and Table 1). Most patients (86%) were treated in the ED and discharged within 24 h of admission; the remaining 24% were hospitalized for poor general conditions, severe symptoms, poor response to supportive treatment, and/or CPK > 4000. Among them, fifteen patients (23%) were admitted to the Pediatric ward, whereas only 1 patient (1.53%) was admitted to the Pediatric Neurology ward; no one needed Pediatric Intensive Care Unit (PICU) admission either at onset or during hospitalization. Among hospitalized patients, the median LOS was 5 days (IQR 3–7.25).

**Table 1:** Epidemiological data and clinical manifestations

**BACM cases**, ***n***	65
**Gender, *n* (%)**	
Male	43 (66.2)
Female	22 (33.8)
**Age, years**	
Median (IQR)	6.5 (4.9–9.1)
**Ethnicity, *n* (%)**	
Caucasian	45 (70)
Asiatic	7 (10.7)
Hispanic	8 (12.3)
African	5 (7)
**Season of admission, *n* (%)**	
Winter (December–March)	45 (69.2)
Spring (March–June)	3 (4.6)
Summer (June–September)	6 (9.2)
Autumn (September–December)	11 (16.9)
**Prodromal symptoms, *n* (%)**	
Fever	49 (75.3)
Cough	21 (32.3)
Coryza	17 (26.1)
Sore throat	17 (26.1)
Nausea	9 (13.8)
Vomiting	10 (15.3)
Asthenia	29 (44.6)
**Presenting symptoms/signs, *n* (%)**	
Bilateral calf pain	65 (100)
Gait complaint	9 (13.8)
Refusing to walk	61 (93.8)
Generalized myalgia	3 (4.6)
Gastrocnemius–soleus tenderness	62 (95.4)

### Clinical manifestations

All patients had bilateral calf pain at admission, most of them (*n* = 57, 87.7%) associated with asthenia and refuse to walk (*n* = 61, 93.8%). Other symptoms at admission and/or during the week before were as follows: fever in 49 children (75.3%), cough in 21 (32.3%), coryza in 17 (26.1%), sore throat in 17 (26.1%), vomiting in 10 (15.3%), tremors in 9 (13.8%), hyperchromic urine in 1 (1.5%) (Table 1). Clinical examination showed in most children tenderness in gastrocnemius–soleus muscles (*n* = 62, 95.4%), with preserved tendon reflexes in all (Table 1). The median time for myalgia resolution was 3 days (IQR 1.75–4). Electrocardiogram (ECG) was performed in 18 patients (27.7%) at admission, and in 3 (4.6%), it revealed repolarization abnormalities that resolved in 3 days.

### Laboratory tests

Laboratory test results of hospitalized and ED-discharged patients are summarized in Table 2. Out of the total number of patients, in 31 (47.6%) of them, laboratory tests were performed only at admission due to a quick discharge from ED. Overall, the median value of CPK was 943 U/L (IQR 577.2–2033.2) at admission and 1827 U/L (IQR 915.5–2462) at peak. LDH showed a median value of 328.5 U/L (IQR 291–380.2) at admission and 363 U/L (IQR 265–438.2) at peak, while AST had a median value of 68.5 U/L (IQR 50.2–118) at the onset and 80 U/L (IQR 65.7–132) at nadir. CPK and LDH median time to nadir was 1 day from the admission while AST median time to peak was 2 days. CPK median time to normalization was 7 days (IQR 7–8.5) from the nadir. Only one patient (6.25%) showed hyperkalemia at admission (K+ 6.2 mEq/L) that resolved in the first 24 h of hospitalization by intravenous hydration (Table 2). Complete blood count test at admission showed leucocytosis in 20 cases (13%), while leukopenia was found in 47 children (72.3%) with a median value of leucocyte 4150/mmc (IQR 2450–4375). CRP was elevated in 26 patients at admission (40%) with a median value of 2.35 mg/dL (IQR 0.95–4.33). A urine test was performed in all patients; it revealed mild and transitory proteinuria in 4 of them (6.1%).

**Table 2:** Laboratory tests median values and IQR

**Lab parameter**	**Onset** **Median value (IQR)**			**Peak** **Median value (IQR)**			**Time to peak** **Median value (IQR)**
	**ED**	**Hospitalized**	**All**	**ED**	**Hospitalized**	**All**	**All**
CPK (U/L)	717.5 (469.7–1399.2)	1707 (1151.2–3620.2)	943 (577.2–2033.2)	1575 (592–1845.5)	2183.5 (1395.5–7156.2)	1827 (915.5–2462)	1 (1–2)
LDH (U/L)	312 (254.7–3492)	441.5 (338.5–662.7)	328.5 (291–380.2)	380 (285.5–423.5)	432 (346–717.5)	363 (265–438.2)	1
AST (U/L)	57 (46.5–74)	153 (86–239.5)	68.5 (50.2–118)	74 (67–96)	153 (86–263)	80 (65.7–132)	2 (1–2.5)
ALT (U/L)	24 (20–28)	45.5 (25.7–107.2)	28 (20–44.5)	38 (33–47)	55.5 (33.5–133.5)	34 (30–47)	2 (1–2.2)
Urea (U/L)	25 (20–28)	24 (16.7–27.5)	24.5 (20–28.2)	17 (14–23)	24.5 (19.2–28.2)	24.5 (20–28.2)	1 (1–2)
Potassium (mEq/L)	4	4	4	4 (3.3–4.6)	4	4	1 (1–3)

### Etiology

**Fig. 3:**
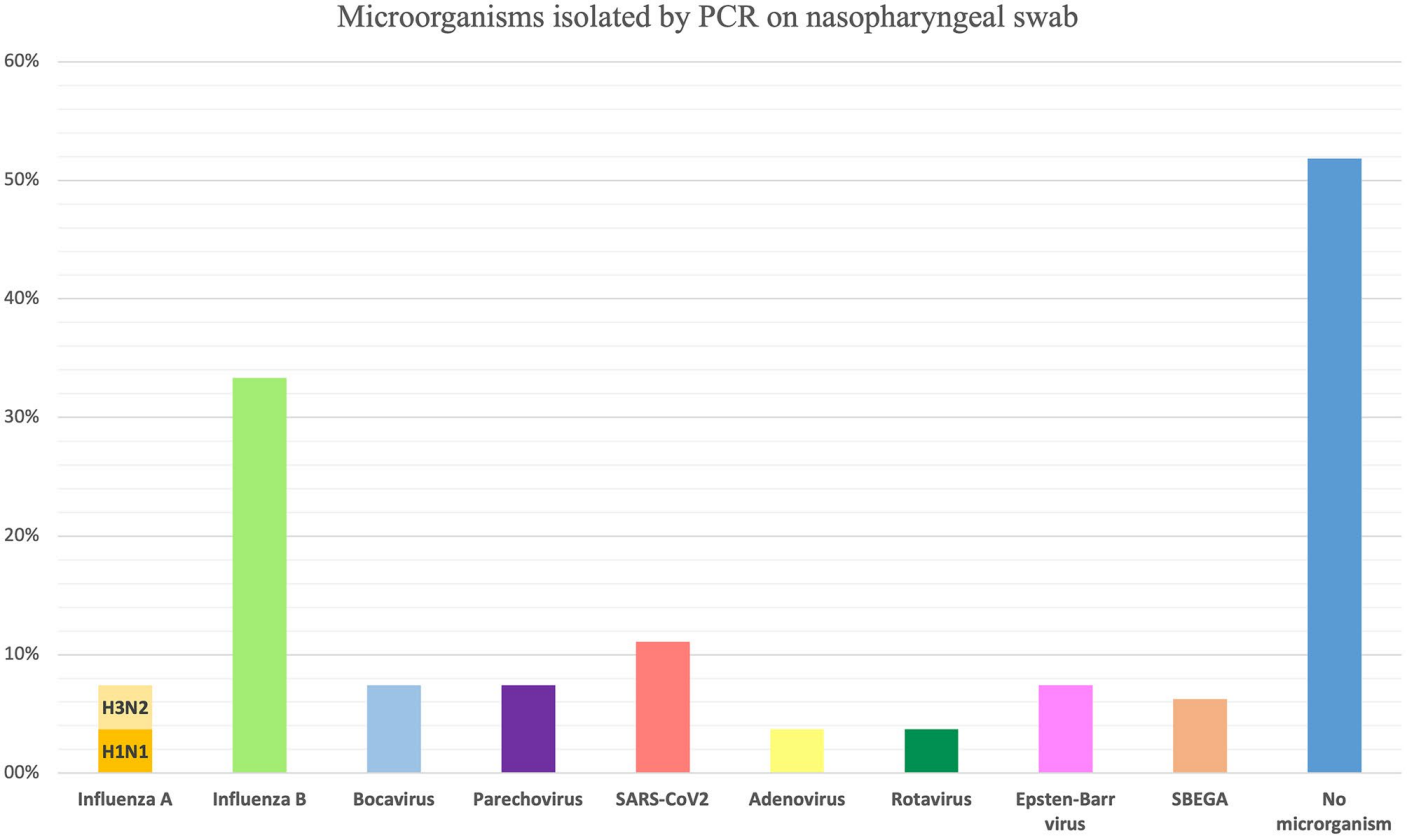
Microorganisms isolated by PCR on nasopharyngeal swab (percentages are on patients who were tested for any infectious agent (*n* = 27))

Real-time Polymerase-chain-reaction (PCR) panel for respiratory viruses on nasopharyngeal swabs was performed in 27 children (41.5%). It was negative for virus detection in 14 patients (21%). The virus most frequently found was the Influenza B virus (9 patients, 13.8%) (Fig. 3). Four patients (6.1%) had more than one micro-organism isolated by nasopharyngeal swab test. One patient was positive for *Streptococcus β haemolyticus group A* (SβGA) rapid test. Two patients (3%) were found positive for EBV infection, tested by both PCR EBV on pharyngeal swab and EBV serology. Rotavirus stool antigen test was found positive in one case (3.70%).

In 6 patients, muscular and metabolic tests such as plasmatic acylcarnitine, plasmatic aminoacidogram, urinary organic acids, and Next Generation Sequencing (NGS) panel for myopathies were performed because of the previous history of hyperCKemia/myositis or due to the extremely high CPK values (≥ 5.000 U/L) at peak. In one patient, the NGS panel revealed RYR1 pathogenic variant causing hyperthermia maligna; this child had a previous history of myositis. In another patient, the NGS panel showed a pathogenic variant in the FKRP gene causing Limb-girdle muscular dystrophy type 1 (LGMD1); this patient had CPK peak values > 17,000 U/L.

### Follow-up

All patients were discharged with a scheduled check-up by their General Pediatrician. Therefore, clinical and biochemical control data are not available. However, none of the patients made new access to the ED for BACM-compatible symptoms in the month following discharge. The two patients with pathogenic variants at the NGS panel for myopathies were subsequently followed by the Neurometabolic and Muscle Diseases Unit.

## Discussion

BACM is a self-limited childhood illness, and viral infections mainly cause it. Most of the patients in our series were preschool-aged male children, a distribution that is congruous with reports in the literature. The reason for the male predominance is unknown, but it could be due to a genetic predisposition [11–14]. The median age (6.5 years) of our patients resulted slightly higher than the median age reported by some previous studies [3, 11, 15, 16]. Our case series show a noticeable increase in the number of BACM cases per year following the suspension of social distancing measures when comparing the pre- and post-COVID-19 pandemic period. Otherwise, no differences were found in the clinical and/or biochemical presentation of BACM cases between the two periods. Most of the cases occurred in January and February, followed by March and December; this is partially consistent with some larger previous studies, which found most cases in late autumn followed by winter and early spring [3, 11, 15, 17]. Otherwise, Öztürk et al. and Turan et al. included only flu season cases in their studies [3, 17] while Costa Azevedo et al. and Brisca et al. [13, 15] analyzed BACM cases over the pre-COVID years, 2015–2019 and 2010–2018 respectively. Therefore, the partial discordance of our series could be attributed to virus infection seasonality changes during and after the Sars-CoV-2 outbreak. In our study, the first cause of BACM was Influenza virus infection; in particular, Influenza B virus has been found in most cases, which is like with literature [3, 11, 15, 17, 18]. We did not find any case related to *Mycoplasma pneumoniae* infection, unlike with results reported by D’amico et al., who found it in 4% of cases [16]. Furthermore, three of our patients were diagnosed with COVID-19, an emerging cause of BACM. Indeed, previous studies reported that 11–35% of the patients infected with SARS-CoV-2 had muscle involvement [18]. Infectious investigations were negative in one-fifth of patients; nevertheless, all presented upper-airways infection symptoms during the days preceding myositis onset. Failure to identify the viral agent also occurred in all previous case series with a rate ranging from 5.3% [17] to 66% [16]. The clinical presentation of our children was typical: acute onset of symmetrical calf muscle pain that results in the inability or refusal to walk after a flu-like illness. The most common prodromal symptoms were fever, cough, and rhinorrhea, while gastrointestinal symptoms were rare, according to previous case series [3–5, 7, 11, 14]. Gait complaints and refusal to weight bear were more frequent in our cohort than in the larger previous studies [15, 17]; however, it is not possible to make an objective comparison since these types of manifestations differ based on the age and compliance of the pediatric patients. The most striking laboratory finding was the markedly elevated CPK level, which indicates muscle damage and typically normalizes in 1 week. In our study, the median value of CPK at the peak was 1827 UI/L, mostly consistent with median values reported in some previous studies [3, 11, 13, 18]. Contrarily, in the large series of Brisca et al., a lower median value of CPK (1413 UI/L) was found, but the authors considered only CPK values at BACM onset [15]. On the other hand, in the Turkish cohort by Turan et al., the CPK median value was higher (3332 UI/L); the authors, however, reported in their series, a rate of rhabdomyolysis and metabolic diseases (3% and 3.5%, respectively) conditions usually characterized by very high muscles enzymes levels [17]*.* As in our study, Turan et al. performed metabolic screening in all recurrent cases of BACM and/or those with serum CK ≥ 6000 IU/L; three cases were diagnosed with long-chain fatty acid oxidation disorder (LCFAOD) and one with very long-chain acyl-CoA dehydrogenase deficiency (VLCADD). In our cohort, muscular and metabolic tests (acylcarnitine, plasmatic aminoacid-gram, and NGS myopathies panel) were performed in 9.2% of patients; the NGS panel allowed the diagnosis of neuromuscular disorders in two cases, one with LGMD1 and the other with RYR1 pathogenic variant for hyperthermia maligna. The first had a CPK peak value (17,690 UI/L) significantly higher than the median values of the cohort with a normalization time significantly longer (8 days) than the others; the second, with RYR1 mutation and COVID infection, had a history of two previous episodes of myositis. As reported in the literature regarding the increase of the other muscle enzymes [3, 11, 17], AST increase was higher than ALT, and it was found in > 80% of BACM together with LDH elevation also in our cohort. AST, ALT, and LDH showed a steady and significant declining trend till the 5th day of the hospital stay. Regarding the blood count cells, leukopenia was the most common alteration, either with neutropenia and/or lymphopenia. This result supports the viral etiology of BACM, and it is in line with those found in previous reports [7, 11, 19, 20]. None of the patients required more than supportive treatment in the form of paracetamol and fluids. Patients were discharged after the resolution of BACM symptoms, and all patients made a rapid recovery with a median of 5 days without any sequelae. Unlike the other larger previous studies, Turan et al. reported a rhabdomyolysis prevalence of 3% in their series, with 8 cases of acute kidney injury (AKI) and two patients admitted to PICU [17]. However, most literature data support that patients affected by BACM could be effectively managed with analgesia, rest, and adequate hydration at home with clinical follow-up. A full clinical and laboratory recovery can be expected after 1 − 2 weeks. We agree with this point, but we also recommend hospitalization if oral rehydration is not possible and in the case of clinical and laboratory findings compatible with rhabdomyolysis and/or at risk for AKI, as reported in Brisca et al. BACM diagnostic pathway [15].

### Study limitations

This study is limited by the retrospective approach and the limited number of patients and study period. A further limitation is the heterogeneity of the observation period between ED-discharged patients and those hospitalized. To better characterized pediatric myositis and distinguish predictor factors of benign evolution compared to other forms of myositis, prospective studies are needed.

## Conclusions

School-aged children admitted to the hospital with walking difficulty generally after an upper respiratory tract infection with a moderate creatine kinase elevation should remind at first of acute benign myositis. Endemic and pandemic infections may cause this entity as well. A detailed history and thorough physical exam should be performed to distinguish the accompanying features of BACM from other neuromuscular etiologies. Resolution of the complaints in a short time and normalization of the biochemical markers will prevent unnecessary tests. Muscular and metabolic investigations might be considered in recurrent forms and in the case of severe hyperCKemia or rhabdomyolysis. When oral hydration is possible, in uncomplicated forms, outpatient management is possible and recommended.

## Data Availability

The data supporting this study's findings are available on request from the corresponding author, F.A. The data are not publicly available due to privacy and ethical restrictions.

## Abbreviations

AKI: Acute kidney injury
ALT: Alanine aminotransferase
AST: Aspartate aminotransferase
BACM: Benign acute childhood myositis
CPK: Creatine phosphokinase
CRP: C-reactive protein
EBV: Epstein-Barr virus
ECG: Electrocardiogram
ED: Emergency Department
ICD-9-CM: International Classification of Diseases, 9th Revision, Clinical Modification
IQR: Interquartile range
LCFAOD: Long-chain fatty acid oxidation disorder
LDH: Lactate dehydrogenase
LGMD1: Limb-girdle muscular dystrophy type 1
LOS: Length of stay
NGS: Next Generation Sequencing
PCR: Real-time Polymerase-chain-reaction
PICU: Pediatric intensive care unit
SβEGA: Streptococcus β haemolyticus group A
VLCADD: Very long-chain acyl-CoA dehydrogenase deficiency
